# Hydrogen: A Potential New Adjuvant Therapy for COVID-19 Patients

**DOI:** 10.3389/fphar.2020.543718

**Published:** 2020-10-15

**Authors:** Fuxun Yang, Ruiming Yue, Xiaoxiu Luo, Rongan Liu, Xiaobo Huang

**Affiliations:** Department of Critical Care Medicine, Sichuan Provincial People's Hospital, Chengdu, China

**Keywords:** Hydrogen, COVID-19, therapy, cytokine storm, mucus

## Abstract

Hydrogen has been shown to have antioxidant, anti-inflammatory, hormone-regulating, and apoptosis-resistance properties, among others. Based on a review of the research, the use of hydrogen might reduce the destructive cytokine storm and lung injury caused by SARS-CoV-2 during COVID-19 (Corona Virus Disease 2019) in the early stage, stimulating ropy sputum drainage, and ultimately reducing the incidence of severe disease. Molecular hydrogen treatment has the potential to become a new adjuvant therapy for COVID-19, but its efficacy and safety require large clinical trials and further confirmation.

## Introduction

Since the Corona Virus Disease 2019 (COVID-19) was first reported in Wuhan at the end of December 2019, it quickly became the sixth-largest public health emergency and a matter of international concern (Lai et al., 2020). Up to 11:00 on July 31, 2020, there were 17,328,002 confirmed cases in the world, and the cumulative number of deaths was 670,287 with an overall mortality rate of 3.8% (https://covid19.who.int/). Moreover, there is no specific antiviral drug or vaccine that could be used to prevent COVID-19. Huang et al. (2020) found that the plasma concentrations of IL-2, IL-7, IL-10, and TNF-α in severe or critical patients were higher than those in other patients. This is consistent with the pathological findings of Wang Fushen (Liu et al., 2020; Xu Z. et al., 2020). Therefore, Chen et al. propose that cytokine storm is one of the most important factors of the disease in critically ill patients (Chen et al., 2020). Currently, there is no specific drug that can be used to treat cytokine storms.

Hydrogen is a colorless, odorless, and tasteless gas. In 2007, Ohsawa et al. (2007) published a paper in *Nature* outlining that the inhalation of 2% hydrogen could selectively eliminate hydroxyl radical (OH) and peroxynitrite anion (ONOO−) and significantly improve cerebral ischemia-reperfusion injury in rats, which has initiated an upsurge in molecular biology research based on hydrogen. To date, the biological effects of hydrogen have been widely studied. Based on its biological effects, such as in anti-oxidation, anti-inflammation, anti-apoptosis, and hormone regulatory, it has been established that hydrogen has protective effects against a variety of diseases. In particular, the small molecular properties of hydrogen ensure that it quickly reaches the alveoli, which suggests a unique advantage for lung diseases. Given the current epidemic and based on clinical experience, safety, operability, and simple clinical promotion, this review discusses the feasibility of hydrogen as a means of controlling and preventing COVID-19.

## Hydrogen and the Cytokine Storm

Immune cells can become activated, producing pro-inflammatory cytokines, including tumor necrosis factor-α (TNF-α), interleukins (such as IL-1β and IL-6), and interferon-γ (IFN-γ) (Taniguchi and Karin, 2018). An effect of cytokines is the activation of the NADPH oxidase in leukocytes, which leads to the production of reactive oxygen species (ROS) such as superoxide, hydroxyl radicals, and singlet oxygen (Liu et al., 2015). In 1993, Ferrara et al. first proposed the concept of a cytokine storm in graft-versus-host disease (Ferrara et al., 1993). SARS coronavirus infection was found to induce an interferon-γ-related cytokine storm, which might be related to the immunopathological damage observed in SARS patients (Huang et al., 2005). In 2005, a study on avian influenza A H5N1 suggested that high viral loads and the resulting intense inflammatory response are key to its onset (de Jong et al., 2006). Cytokine storms have also been reported in influenza (Kalil and Thomas, 2019) and Middle East respiratory syndrome (MERS) (Channappanavar and Perlman, 2017). At present, the factor that causes cytokine storms is not clear, but it is generally believed that the immune system overreacts to new and highly pathogenic pathogens. The relating imbalance of the immune regulatory network, the lack of negative feedback, and the continuous self-amplification of positive feedback lead to an abnormal increase in many kinds of cytokines, and finally to a cytokine storm. Although the pathophysiological mechanism underlying COVID-19 is not completely understood, it has been reported that there are large quantities of cytokines such as IL-1 β, INF-γ, IP-10, and MCP-1 in COVID-19 patients, which might activate Th1 cells. The concentrations of G-CSF, IP-10, MCP-1, MIP-IA, and TNF-α in critically ill patients were found to be higher than those in non-critical patients, indicating that cytokine storms might be related to the severity of the disease (Liu et al., 2020). The effectiveness of anti-IL6-receptor and glucocorticoid therapy for patients with COVID-19 was only verified in a small number of patients (Selvaraj et al., 2020; Xu X. et al., 2020). However, more clinical studies are underway with respect to treating COVID-19 with tocilizumab and dexamethasone (NCT04445272, NCT04244591, NCT04381364). Corticosteroids suppress lung inflammation but also inhibit immune responses and pathogen clearance (Russell et al., 2020). Furthermore, the use of anti-IL6-receptor therapy for patients with rheumatic diseases might lead to an increased risk of infection (Rutherford et al., 2018). Due to these potential side effects, tocilizumab and dexamethasone have not been widely used in clinical practice.

Cytokines released excessively can cause acute lung injury in patients. An increase in TNF-α levels will lead to the activation of inflammatory cytokines such as IL-1, IL-6, and IL-8 (Chen et al., 2015). At the same time, high mobility group box1 (HMGB1) (Ma et al., 2015), CCL2 (Hillman et al., 2007), and Egr-1 (Hoetzel et al., 2008) all affect the release of inflammatory factors. Keliang Xie found that hydrogen can suppress the infiltration of neutrophils and macrophages in lung tissue, inhibit the activity of NF-κB and MPO in lung tissue, and reduce inflammatory factors and cytokine secretion in lung tissue, including TNF-α, IL-1, IL-6, and HMGB1. Hydrogen can eliminate ROS, such as hydroxyl and peroxynitrate anions, while maintaining the normal metabolism of redox reactions and other ROS (Xie et al., 2012). Accordingly, hydrogen treatment can reduce the levels of TNF-α, IL-1, IL-1 β, IL-6, IL-8, HMGB1, CCL2, and Egr-1 in lung tissue in an animal model (Huang et al., 2010a). Furthermore, inhaling hydrogen for 45 minutes can reduce airway inflammation in patients with asthma and COPD (Wang et al., 2020). At the same time, previous studies have shown that an increase in IL-10 can inhibit the synthesis and release of inflammatory cells and colony stimulating factors (Laveda et al., 2006). After inhaling hydrogen, IL-10 was found to increase in the serum and sputum supernatant of sanitation workers (Gong et al. 2016), indicating that this treatment can affect anti-inflammatory reactions and reduce secondary injury caused by cytokine storms. Some critical patients with pneumonia need to be supported by mechanical ventilation. However, this can cause lung injury or aggravate the original lung injury. In a rat model of mechanically ventilated lung injury, Huang et al. (Huang et al., 2010a) found that after inhaling 2% hydrogen the expression of NF-kappa B was activated, promoting the expression of the anti-apoptotic protein Bcl-2, inhibiting expression of the apoptotic protein Bax, suppressing inflammatory factor expression, decreasing the lung histopathological score, and alleviating pulmonary edema, thus diminishing ventilator-related acute lung injury. In addition, hydrogen can inhibit the Rho/ROCK pathway, increase the expression of ZO-1, and protect lung tissue cells by improving cell-to-cell permeability, and reducing lung injury (Zhang et al., 2016). Therefore, the early use of hydrogen in COVID-19 patients could potentially suppress the release of cytokines and reduce lung injury.

## Hydrogen and Oxidative Stress Reactions in COVID-19

Superoxide dismutase (SOD) is an important antioxidant enzyme in the antioxidant defense system of the body. It can remove a variety of toxic or oxidizing substances in the body to eliminate the damage to DNA and biological functional proteins caused by these substances to maintain the stability of the internal environment and contribute to anti-toxicity and anti-oxidation processes (Gwarzo and Muhammad, 2010). After hydrogen treatment, the content of malondialdehyde in lung tissue can be reduced, increasing the activity of SOD (Shi et al. 2013). This helps to maintain the stability of the internal environment of the body to achieve excessive activation of oxidative processes and reduce the oxidative stress caused by the ROS pathway. Multiple organ failure is a common cause of death in critically ill COVID-19 patients. Hydrogen may be used to protect multiple organs including the heart, kidney, and nervous system *via* anti-apoptotic and anti-oxidative functions to maintain the normal response of the body and reduce mortality (Hayashida et al., 2012; Hayashida et al., 2014; Homma et al., 2014).

## Hydrogen Reduces COVID-19-Related Viscous Secretions

Based on the results of pathological anatomy, Liu Liang’s team found that in addition to excessive inflammatory reactions, many viscous secretions that spilled from the alveoli and fibrous cords could be seen in lung sections, and the viscous secretions were mainly concentrated in the terminal bronchi (Liu et al., 2020). This is inconsistent with the clinical manifestation of dry cough without sputum. Clinical oxygen therapy is delivered mainly *via* nasal high-flow oxygen inhalation and non-invasive ventilator-assisted ventilation. Accordingly, its positive pressure ventilation mode will cause the accumulation of distal bronchial viscous secretions, increase airway resistance, alter the effect of oxygen therapy, and aggravate systemic hypoxia. This finding implies new thinking that could adjust the regimen used in clinical treatment. Drug atomization and humidification might become indispensable treatment methods, but in terms of the treatment process, attention should be paid to the third-level protection of medical staff to prevent aerosol transmission, which would increase the risk of infection. Mucus is composed of water, ions, lipids, proteins, and complexes (Voynow et al., 2006). In an animal model, airway mucus was found to play an important role in the host defense mechanism, but the production of excessive mucus is harmful (Shimizu et al., 2012). Muc5ac and Muc5b are the components of mucin, and Muc5ac is produced by goblet cells among airway epithelial cells (Perezvilar et al., 2006). Rats treated with hydrogen-enriched water had reduced airway damage, Muc5ac expression, and mucus secretion in smog-induced COPD models (Ning et al., 2013). Therefore, early hydrogen inhalation may promote sputum dilution, improve small airway resistance, and relieve dyspnea.

## Safety of Hydrogen

Based on a clinical hydrogen test, hydrogen absorption and drinking hydrogen-rich water are used for treatment. The potential anti-fatigue and performance benefits of hydrogen rich water (HRW) have received increased research interest over the past 5 years. For example, acute pre-exercise supplementation with HRW reduces blood lactate at higher exercise intensities, improves the exercise-induced perception of effort, and increases ventilatory efficiency (Botek et al. 2019). At the same time, hydrogen, as a flammable and explosive small molecular substance, has been developed clinically and can be safely applied with access to medical devices. Clinical research has shown that hydrogen dissolved in irrigation solution reduces corneal endothelial damage during phacoemulsification (Igarashi et al., 2019). Furthermore, breathing H_2_-O_2_ might reduce the inspiratory effort in patients with acute severe tracheal stenosis and can be used safely for this purpose (Zhou et al., 2019). Although H_2_ gas is flammable, concentrations < 4%, together with oxygen at room temperature, are incombustible. As indicated by the second law of thermodynamics, although several physical processes that satisfy the first law are possible, the only processes that occur in nature are those for which the entropy of the system either remains constant or increases. Thus, exhaled H_2_ diffuses instantly, without accumulating or resulting in an increased concentration exceeding the inspiratory H_2_ concentration. Therefore, 2% H_2_ gas can be carefully administered in a hospital (Tamura et al., 2017). During this treatment, a very small number of patients have symptoms such as sparse stool, increased defecation frequency, heartburn, and headache after drinking. These symptoms may be relieved without intervention after the patient stops hydrogen treatment. At the same time, these symptoms have no serious adverse events, and the associated harm is minimal (Atsunori et al., 2010; Huang et al., 2010b).

## Conclusions

In summary, we hypothesize that the early use of hydrogen might mitigate the destruction caused by the cytokine storm associated with COVID-19, reducing lung injury, promoting viscous sputum drainage, and thus reducing the incidence of critically ill patients. Only one other article to date has mentioned the use of hydrogen to treat COVID-19 patients (Guan et al., 2020). In the future, more large-scale randomized controlled trials are needed to verify the efficacy and safety of this treatment clinically.

## Author Contributions

FX Y, RM Y, and RA L wrote the original draft. XL undertook validation, writing, review, and editing. XH undertook writing, review, and editing. All authors contributed to the article and approved the submitted version.

## Fundng

This study was supported by the Sichuan Province Science and Technology Support Program (numbers: 2017SZ0138)and Chengdu Science and Technology Support Program (numbers: 2020-YF05-00104-SN).

## Conflict of Interest

The authors declare that the research was conducted in the absence of any commercial or financial relationships that could be construed as a potential conflict of interest.
